# Supplementary material for: Chemical, Pharmacological and Computerized Molecular Analysis of Stem’s Extracts of Bauhinia scandens L. Provide Insights into the Management of Diarrheal and Microbial Infections
Source: Nutrients. 2022 Jan 9;14(2):265. doi: 10.3390/nu14020265 (PMC8778859; doi:10.3390/nu14020265)
Supplement: Supplementary file 1 [file nutrients-14-00265-s001.zip › nutrients-1520213-supplementary.pdf]

Table S1. Identified flavonoids in the methanol extract of the *Bauhinia scandens* by chromatography (UPLC-QTOF–MS).

Component name	Formula	Identification status	Observed neutral mass (Da)	Observed m/z	Mass error (mDa)	Mass error (ppm)	Observed RT (min)	Response	Adducts	Observed CCS (Å²)	Total Fragments Found
Moracin M-3′-O-β-D- glucopyranoside	C20H20O9	Identified	404.1136	427.1028	2.8	6.7	0.50	1750	+Na	224.38	9
Brazilein	C16H12O5	Identified	284.0708	291.0863	2.4	8.1	0.51	1161	+Li	225.22	5
Forsythoside D	C20H30O13	Identified	478.1689	501.1581	0.3	0.6	0.53	1823	+Na, +K	204.53	0
Koaburaside	C14H20O9	Identified	332.1107	355.0999	-0.1	-0.2	2.81	1177	+Na	174.07	0
Decaffeoylacteoside	C20H30O12	Identified	462.1741	485.1633	0.4	0.8	4.84	1248	+Na	200.42	0
Methyl-5-O-Caffeoylquinate	C17H20O9	Identified	368.1082	369.1155	-2.5	-6.8	4.99	1284	+H	182.14	0
Meliadanoside A	C16H24O10	Identified	376.1381	377.1454	1.2	3.1	6.01	1812	+H	184.66	1
2′-Hydroxy-3′,4′- dimethoxyisoflavan-7- O-β-D-glucoside	C23H28O10	Identified	464.1684	471.1838	0.1	0.3	6.23	1428	+Li	212.80	1
Dendroflorin	C14H10O5	Identified	258.0527	259.0599	-0.2	-0.7	7.36	1159	+H	154.34	3
Blestrianol B	C37H32O7	Identified	588.2186	589.2259	3.8	6.5	8.33	77522	+H	228.71	7
Dihydroresveratrol	C14H14O3	Identified	230.0941	231.1014	-0.2	-0.8	8.34	1271	+H	148.90	0
4′-Methylpinosylvin	C15H14O2	Identified	226.1015	233.1169	2.1	8.9	8.34	1393	+Li	147.65	0
Gigantol	C15H16O4	Identified	260.1069	267.1223	2.0	7.5	8.34	1242	+Li	229.90	1
Lusianthridin	C15H14O3	Identified	242.0964	249.1119	2.1	8.5	8.63	2717	+Li	151.14	1
Blestrianol D	C29H24O5	Identified	452.1600	453.1673	-2.4	-5.2	8.76	1062	+H	183.20	0
Dihydrocurcumin	C21H22O6	Identified	370.1415	371.1488	-0.2	-0.4	8.78	2648	+H	185.85	7
Protosappanin A	C15H12O5	Identified	272.0674	273.0747	-1.1	-3.9	8.98	5570	+H	160.75	4
(3R)-3′,8-Dihydroxyvestitol	C16H16O6	Identified	304.0925	305.0998	-2.2	-7.2	9.09	4978	+H	160.65	1
Chrysotoxine	C18H22O5	Identified	318.1492	341.1384	2.5	7.3	9.11	1804	+Na	177.42	0
2,3,5,4'-Tetrahy-	C26H32O14	Identified	568.1797	591.1689	0.5	0.8	9.32	1142	+Na	222.51	0

droxystil-bene-2- O-(6''-O-α-D-glucopy-ranosyl)-β-D-glucopy-ranoside											
Mulberro-furan F	C39H34O8	Identified	630.2293	631.2366	3.9	6.2	9.41	4869	+H	241.05	0
Xantho-humol	C21H22O5	Identified	354.1470	355.1543	0.3	0.8	9.46	1178	+H	182.48	3
4,5-O-Dicaffeoyl quinic acid	C25H24O12	Identified	516.1287	555.0919	1.9	3.5	9.87	3290	+K	215.95	0
2-Octylphe-nol	C14H22O	Identified	206.1690	229.1582	1.9	8.3	10.07	1218	+Na	196.69	0
3,4-O-Dicaffeoyl quinic acid	C25H24O12	Identified	516.1290	555.0922	2.3	4.1	10.33	4025	+K	229.15	0
3,7-Dihy-droxy-2,4-dimethox-yphenan-thren e-3-O-glucoside	C22H24O9	Identified	432.1449	455.1341	2.9	6.3	10.73	1356	+Na	213.02	1
Cyclocur-cumin	C21H20O6	Identified	368.1257	369.1330	-0.3	-0.7	11.87	1250	+H	191.08	10
10-O-Methylpro tosappani n B	C17H18O6	Identified	318.1075	319.1147	-2.9	-9.0	13.12	2235	+H	167.04	0
Apocynin B	C24H20O10	Identified	468.1034	469.1107	-2.2	-4.8	13.21	1308	+H	196.84	0
Mulberro-furan K	C39H32O8	Identified	628.2134	629.2207	3.7	5.8	13.26	1431	+H	228.22	0
Pro-tosappani n C	C16H14O6	Identified	302.0788	303.0861	-0.2	-0.7	13.30	10492	+H	167.33	4
Caesalpins P	C16H12O6	Identified	300.0635	301.0708	0.1	0.5	13.38	9583	+H	162.68	2
Gingerone	C11H14O3	Identified	194.0939	195.1011	-0.4	-2.2	13.45	1036	+H	142.31	0
Confusar-in	C17H16O5	Identified	300.0994	301.1067	-0.3	-1.1	13.67	2392	+H	174.95	1
Nobilin D	C16H18O6	Identified	306.1126	329.1019	2.3	7.0	13.67	1810	+Na	177.24	2
Dendro-candin E	C15H16O5	Identified	276.1020	299.0912	2.2	7.4	13.78	1162	+Na	241.80	0
Euparin	C13H12O3	Identified	216.0810	239.0702	2.3	9.7	13.88	7860	+Na	148.59	0
Blestritin B	C30H30O6	Identified	486.2075	487.2148	3.3	6.7	13.89	25929	+H	202.17	0
Moracin B	C16H14O5	Identified	286.0838	287.0911	-0.3	-1.0	13.93	1609	+H	162.51	1
Cin-chonain Ia	C24H20O9	Identified	452.1109	453.1181	0.1	0.3	14.13	1076	+H	209.49	1

Kuzubu-tenolide A	$C_{23}H_{24}O_{10}$	Identified	460.1351	461.1424	-1.8	-4.0	14.29	1497	+H	200.12	1
Forsy-thoside B	$C_{34}H_{44}O_{19}$	Identified	756.2452	795.2083	-2.5	-3.1	14.94	1308	+K	219.34	2
Asebotin	$C_{22}H_{26}O_{10}$	Identified	450.1559	473.1451	3.3	6.9	14.94	1891	+Na	280.61	1
3,7-Dihy-droxy-2,4-dimethox-yphenan-thren e-3-O-glucoside	$C_{22}H_{24}O_9$	Identified	432.1439	455.1331	1.9	4.2	14.94	6938	+Na	277.86	1
2,5-Dihy-droxy-4,9-dimethox-yphenan-threne	$C_{16}H_{14}O_4$	Identified	270.0887	271.0960	-0.5	-2.0	15.05	4461	+H	160.58	
Protohy-pericin	$C_{30}H_{18}O_8$	Identified	506.0999	507.1072	-0.3	-0.5	15.08	1545	+H	217.37	0
Brazilin	$C_{16}H_{14}O_5$	Identified	286.0838	287.0911	-0.3	-1.0	15.41	28734	+H	162.09	2
Dendro-candin B	$C_{27}H_{30}O_8$	Identified	482.1975	483.2048	3.5	7.2	15.59	1092	+H	257.34	0
Smilaxin	$C_{17}H_{16}O_6$	Identified	316.0943	317.1016	-0.4	-1.3	15.73	29199	+H	172.40	3
Caesalpins J	$C_{17}H_{16}O_6$	Identified	316.0946	317.1018	-0.1	-0.4	15.94	151920	+H	172.14	13
(R)-Pre-chrysoph-anol	$C_{15}H_{14}O_4$	Identified	258.0910	265.1064	1.8	6.7	16.39	1056	+Li	157.54	0
Dendro-candin C	$C_{16}H_{18}O_5$	Identified	290.1160	313.1052	0.6	1.8	16.56	1683	+Na	168.28	1
Caffeate	$C_9H_8O_4$	Identified	180.0416	181.0488	-0.7	-3.8	16.56	1527	+H	131.36	0
2′-Hydroxy-7,3′,4′-tri-methoxy-isoflavan	$C_{18}H_{20}O_5$	Identified	316.1332	323.1487	2.1	6.6	16.60	1182	+Li	213.21	0
Nobilin B	$C_{17}H_{20}O_6$	Identified	320.1281	343.1173	2.1	6.2	16.63	8103	+Na	177.13	5
Feralolide	$C_{18}H_{16}O_7$	Identified	344.0896	345.0969	0.0	0.0	16.63	155181	+H, +Li	175.16	11
2,5-Dihy-droxy-4,9-Di-methoxy-phenan-threne	$C_{16}H_{14}O_4$	Identified	270.0892	271.0965	0.0	-0.1	16.64	10513	+H	159.56	4
3'-O-Methylbrazilin	$C_{17}H_{16}O_5$	Identified	300.0993	301.1066	-0.5	-1.6	16.64	12453	+H	167.24	6
Tor-achrysone-8-O-β-D-glucopy-ranoside	$C_{20}H_{24}O_9$	Identified	408.1433	409.1506	1.3	3.1	16.64	1547	+H	237.62	12

Dihydrooxyresveratrol	C14H14O4	Identified	246.0912	269.0804	1.9	7.2	16.65	5145	+Na, +H	154.21	0
Polydatin	C20H22O8	Identified	390.1341	413.1233	2.6	6.4	16.66	3826	+Na	249.38	14
Albaspidin AA	C21H24O8	Identified	404.1498	427.1390	2.7	6.2	16.68	4884	+Na	243.85	6
Meliadanoside B	C15H20O8	Identified	328.1125	335.1279	-3.3	-9.9	16.75	1515	+Li	178.17	2
